# Genetic Variability in the *CPA1* Gene and Its Impact on Acute Pancreatitis Risk: New Insights from a Large-Scale Study

**DOI:** 10.3390/ijms252011301

**Published:** 2024-10-21

**Authors:** Stanisław Głuszek, Wioletta Adamus-Białek, Magdalena Chrapek, Anna Dziuba, Julia Dulębska, Dorota Kozieł, Jarosław Matykiewicz, Monika Wawszczak-Kasza

**Affiliations:** 1 Department of Surgical Medicine with the Laboratory of Medical Genetics, Collegium Medicum, Jan Kochanowski University of Kielce, 25-317 Kielce, Polandwioletta.adamus-bialek@ujk.edu.pl (W.A.-B.);; 2 Department of Mathematics, Jan Kochanowski University of Kielce, 25-406 Kielce, Poland

**Keywords:** acute pancreatitis, carboxypeptidase A1, genetic risk factor

## Abstract

Acute pancreatitis (AP) is a common and potentially lethal disease. Over the last 10 years, AP has become one of the most important healthcare problems. On a global scale, the incidence has increased by 63% over the last 20 years. AP is usually caused by gallstones and excessive alcohol consumption and genetic factors play an important role in the development of inflammation. Recent studies involving the *CPA1* mutations are ambiguous and dependent on the population studied. In this study, the variability of the *CPA1* gene in patients with AP was analyzed. Genetic material was isolated from the blood of 301 patients with AP and 184 healthy individuals. Identification of the variants in exons 5, 6, 8, and 9 with introns was performed using molecular biology methods. Mutations were identified by comparison to the reference sequence (NM_001868.4). Statistical analysis included the identification of mutations correlating with the risk of AP, the etiology of inflammation, and family history. Several novel mutations in the *CPA1* gene have been identified, along with a high degree of variability within the coding region of the carboxypeptidase gene. A correlation between mutations *CPA1*:c.1072 + 84del; c.987 + 57G>A and increased risk of developing AP was found. Two protective mutations, *CPA1*:c.625A>T, c.1072 + 94del, were identified. The *CPA1* gene is characterized by high sequence variability and regions in which mutations lead to an increased risk of developing AP. Single or co-occurring mutations of the *CPA1* gene can significantly affect the risk of developing AP.

## 1. Introduction

Acute pancreatitis (AP) is a common inflammatory disease, affecting 13 to 45 people per 100,000 in the Eastern European population [1,2]. Over the last ten years, AP has become one of the most important problems related to digestive system disorders, and the number of cases has increased in Europe, Asia, and North America [3,4,5]. It should be emphasized that acute pancreatitis has a significant mortality rate, ranging from 10 to 40% of cases, depending on the severity of pancreatitis [6,7]; therefore, risk factors and individual predispositions should be analyzed. The correlation between gender and acute pancreatitis differs depending on the etiology. In general, AP is diagnosed with the same frequency in women and men but the mortality rate is higher in men. Additionally, the recurrence of the AP is more common in men, who also more frequent develop chronic pancreatitis [8,9]. The well-known etiological factors of AP are gallstones and excessive alcohol consumption, while the disease is less often caused by infectious agents. It is also established that environmental factors and genetic predisposition play an important role in the development and course of acute pancreatitis [10]. To date, variants in *PRSS1*, *SPINK1*, *CFTR*, *CTRC*, *CTSB*, *CASR*, and *TRPV6* have been linked with AP and have a direct impact on the onset of acute pancreatitis [11,12,13,14,15,16,17]. It is worth mentioning the role of gene polymorphisms in *CLDN2* [16] and *CPA1* [18], which are not related to “regulation of intrapancreatic trypsin secretion” but correlate with AP development. The gene encoding carboxypeptidase A1 (*CPA1*) is located on chromosome 7 (7q32.2) in a cluster with genes encoding other pancreatic metalloprotease *CPA2* and those expressed in other tissues (*CPA4* and *CPA5*). Carboxypeptidase A, which is active in the intestine, catalyzes the hydrolysis of C-terminal amino acids in proteins and related esters. In recent years, *CPA1* has attracted significant attention from scientists. There are few reports in the literature on the *CPA1* gene and its role in the development of acute pancreatitis and complications following acute pancreatitis [18,19,20,21]. In addition, the coexistence of mutations in one gene has not been analyzed, which is an innovative aspect of this work. In this study, the variability of the *CPA1* gene in patients with AP was analyzed, including the identification of mutations correlating with the risk of AP, the etiology of inflammation, and family history.

## 2. Results

Variant analysis in exons 5, 6, 8, and 9 was performed for 301 AP patients with different etiologies (data shown in Table 1) and 184 healthy volunteers. The most frequent etiology factor of acute pancreatitis was gallstones in the biliary tract (133/301) followed by alcohol consumption (100/301). Additionally, numerous AP cases were idiopathic (62/301). Family history was analyzed only for AP patients and was positive for 35/256 cases. Among all subjects, 196 mutations were identified, including 84 variants localized in the coding sequence and 112 intron variants. Of these, 86.2% (163/196) of variants were identified in only one analyzed subject. Considering variants in the coding regions, 76 missense mutations, 6 silent mutations, and 2 frameshift variants were identified. All mutations are listed in Supplementary Materials Table S1.

In 98.98% (193/196) of mutations, only heterozygotes were observed. Homozygotes were identified for three mutations: *CPA1*(NM_001868.4)c.696 + 27del, identified in 7/184 controls and 11/301 AP; *CPA1*(NM_001868.4)c.622G>A (p.Ala208Thr), identified in 1/301 AP; and the *CPA1*(NM_001868.4)c.696 + 28insA mutation, identified in 2/301 patients with acute pancreatitis.

Two mutations with higher occurrence in the AP group (increasing the risk of AP) and two with occurrence higher in the control group (protective mutations) were identified. The vast majority of identified variants were located in exon 8 (16.8%) and exon 6 (10.2%). The region *CPA1*(NM_001868.4)c.484 − 33; c.585 + 48 (intron/exon5/intron) was also identified, in which mutations occur more frequently in AP patients (*p* < 0.05). The data are shown in Table 2.

Variants with occurrences close to a statistically significant *p* value (0.06–0.1) were identified (Table 3).

The genotype frequencies in the case of mutation CPA1(NM_001868.4):c.622G>A (p.Ala208Thr) with respect to the family history were also analyzed. No significant differences were observed (Supplementary Materials, Table S2).

## 3. Discussion

### 3.1. Epidemiology of Acute Pancreatitis

In acute pancreatitis, as in the case of many diseases, lifestyle, age, comorbidities, and environmental factors play the most important roles in disease development [22]. Roberts et al. (2017) [4] specified the incidence and etiology of acute pancreatitis in Europe. They clearly showed that in Western Europe (Italy, Greece, Turkey, Spain, Portugal, and Croatia) bile-related AP dominates, being diagnosed up to 10 times more often than alcoholic AP. Completely opposite results were obtained for Eastern Europe, where alcohol-related acute pancreatitis dominates. A 2022 meta-analysis published by Iannuzzi et al. [23] showed that AP affects men and women equally globally. However, studies conducted on the Polish population by Fabisiak et al. [24] indicate a statistically significant difference between men and women. Studies conducted on the Świętokrzyskie Province population by Głuszek et al. confirm that male sex predisposes to AP [2]. In our study, male predominance was also observed in the study group, accounting for 57.8% of the group with acute pancreatitis. A clear association between the etiological factor and gender is observed. In women, biliary AP is diagnosed more often, while in men, alcoholic AP dominates [22]. The same trend was observed in our study group, where biliary AP was found twice as often in women, and alcoholic AP nine times more often in men. Studies published by Roberts et al. [4] show a sudden increase in morbidity after the age of 60. The correlation between the age of patients and the etiological factor indicates that the peak incidence of alcoholic AP is between 35 and 45 years of age, while the biliary etiology is characteristic for the population over 60 years of age. In this study, it was also observed that biliary pancreatitis accounted for 73% of cases over 60 years of age. In the case of alcohol-related AP, the number of cases was similar across all age groups. Acute pancreatitis can also be caused by drug side effects (drug-induced AP). Until 1993, more than 525 drugs were identified that could cause AP. In today’s aging society, drug-induced etiology may be underestimated and may become one of the main causes of pancreatic damage and AP in the future [25,26]. Only two cases of drug-induced AP were identified in this study group.

### 3.2. Specific Mutation Analysis

Family predispositions are strongly related to pancreatitis, both acute and chronic. The main purpose of this research was to assess the variability of the *CPA1* gene sequence in patients with acute pancreatitis diagnosed between 2013 and 2020 in the Świętokrzyskie Province and to determine whether there is a direct relationship between the detected mutations, the risk of the disease, and etiological factors. This study was modeled on research conducted by Witt et al. focusing on regions that correlated the most with the incidence of AP [18]. Mutations in genes encoding pancreatic enzymes, such as *CPA1*, can cause misfolded proteins and endoplasmic reticulum stress, potentially leading to AP. By surveying the extensive region of *CPA1* from exon 5 to 9, along with the flanking intronic regions, significant variation in this area was detected. Many of these variations correlated with clinical data. Notably, two new *CPA1* mutations were identified that were statistically significantly more common in the AP group. These mutations are located in the non-coding sequence and based on the analyses of international databases (NCBI, ClinVar), have not been previously reported. It was estimated that the *CPA1*(NM_001868.4):c.987 + 57G>A mutation doubles the risk of AP [OR = 2.59 [95%CI 1.26;5.32]]. For the *CPA1*(NM_001868.4):c.1072 + 94del deletion, the relative risk and odds ratio were not determined because the mutation was identified only in the AP group. Comparing the loci of the identified mutations with the literature data, it was established that Witt et al. did not record the mutations discovered in this study *CPA1*(NM_001868.4):c.987 + 57G>A and c.1072 + 94del but they identified a mutation in close proximity to *CPA1*(NM_001868.4):c.987 + 58G>A in one control [18]. It should be noted that the studies of Witt et al. were conducted on a larger and more diverse population (Germany, France, Czech Republic, Poland, India, and Japan) [18]. Studies conducted on the Chinese population by Wu H. et al. [20] found no correlation between the *CPA1* variant and the risk of idiopathic chronic pancreatitis. In our study, protective mutations were also detected, including a missense mutation *CPA1*(NM_001868.4):c.625A>T (p.Ile209Phe) and a mutation located in the intron *CPA1*(NM_001868.4):c.1072 + 94del. According to the NCBI database, the mutation at the same location as the *CPA1*(NM_001868.4):c.625A>T (p.Ile209Phe) variant was previously reported (rs782417364) and has no correlation with any disease. The *CPA1*(NM_001868.4):c.1072 + 94del—identified in the intron region—also has not been previously reported. There are no references to the pathogenicity of any of the identified variants in the literature. Protective mutations against pancreatitis have also been described by other authors, such as Witt et al. [27], who described the *PRSS2*(NM_002770.4):c.571G>A (p.G191R) mutation, however, the overall prevalence did not exceed 5%. The protective mechanism was based on the immediate autodegradation of anionic trypsinogen, which protected mutation carriers against the occurrence of CP [27]. The mechanism of influence for the protective variants identified in our study requires further consideration.

### 3.3. General Mutation Analysis

In general, among all identified mutations, one *CPA1*(NM_001868.4):c.622G>A (p.Ala208Thr) was previously described by Witt et al. [18]. Its frequency in the group of Europeans with CP was not statistically significant (7.5% of CP patients and 5.9% of healthy controls). In our study, the frequency of this mutation in patients and healthy volunteers was similar (6.6% and 11%, respectively). For the same mutation, the distribution of genotypes and alleles was determined but no statistical significance was found. In studies conducted by Wu et al. [20] on the Chinese population, this mutation was identified in only one patient with chronic pancreatitis and one healthy person. It can, therefore, be assumed that the frequency of these mutations depends on latitude. In the same study, Wu et al. [20], the occurrence of the p.Gly225Asp mutation was reported in one patient with CP. In our study, this mutation was equally rare in the study group and was identified in one patient with acute pancreatitis. In studies conducted by Witt et al. [18], the p.Gly166Asp mutation was identified in one patient and eleven controls, with no statistically significant differences. This mutation was also identified in our study, however, it occurred only in one patient with acute pancreatitis. Witt et al. [18] identified the p.Tyr308His mutation, which was statistically significantly more frequent in non-alcoholic CP patients (*p* = 0.02). In our study, the p.Tyr308Asn mutation was identified in the same location in one patient with biliary AP.

### 3.4. Consequences of Vatiants In Silico

In silico analysis of all identified mutations for protein synthesis revealed 58 mutations that may affect the amino acid sequence of the nascent CPA1 protein. Each of these mutations has the potential to disrupt enzyme activity or alter protein structure. To confirm these effects, further studies should examine both the level of protein expression and its activity. An unambiguous assessment of the impact of mutations on the occurrence of disease is possible through research using an animal model. Variants that alter the amino acid sequence can lead to the accumulation of misfolded proteins, potentially inducing ERS. The accumulation of misfolded carboxypeptidase can activate an inflammatory response in the pancreas. In 2019, Hegyi E. and Sahin-Toth M. [28] studied in vivo the effect of the *CPA1* p.Asn256Lys mutation on the risk of CP in mice. These were the first studies in a mouse model to determine the impact of misfolded carboxypeptidase protein on the risk of CP and confirm the possibility of ERS-induced inflammation. Studies published by Hegyi E. and Sahin-Toth M. clearly demonstrated that mice carrying the *CPA1* p.Asn256Lys mutation developed chronic pancreatitis. The level of tissue damage increased over time: after three months, 15% of pancreatic tissue was damaged; after 6 months, 20% was damaged; and after a year, about 30% of the pancreatic tissue was damaged. This evidence supports the high potential of the *CPA1* gene mutation as a factor in increasing the risk of developing acute pancreatitis.

### 3.5. Hot Spots of the CPA1 Gene

When analyzing whole gene sequences, it is common to observe that certain regions exhibit a greater accumulation of mutations. For example, Rosendahl et al. [29], Szmola et al. [30], and Kozieł et al. [31] determined that mutations in exons 3 and 7 of the *CTRC* increased the risk of pancreatitis. Consequently, in this study, an attempt was made to identify regions in the carboxypeptidase gene where mutations would increase the risk of AP. The region including exon 5 and part of the adjacent introns *CPA1*(NM_001868.4):c.484 − 33; c.585 + 48 were identified as having high mutagenic potential. It was observed that mutations in this region increase the risk of acute pancreatitis fivefold, despite the largest number of missense mutations being identified in exon 8, followed by exon 6. Comparing the results obtained within the literature data, Witt et al. [18] observed that the greatest number of mutations affecting the secretion and activity of the CPA1 enzyme occurred in the regions of exons 7, 8, and 10. Exons 5, 7, 8, 9, and 10 include amino acids involved in the binding of the zinc ion, which is a cofactor in the enzymatic reaction of CPA1 as well as substrate binding sites. Mutations in this region may therefore result in reduced protein activity or its complete dysfunction.

### 3.6. Coexistence of Mutations—Analysis of Genotypes

In this study, a significant diversity in the number and loci of mutations in the *CPA1* gene was demonstrated across all study groups. As previously mentioned, an area of high mutagenic potential was identified during preliminary analyses. Consequently, innovative research was conducted to select a set of mutations that could indicate a higher risk of AP. The coexistence of configurations in which the number of mutations ranged from 2 to 11 mutations was analyzed. However, no direct correlation was found between the coexistence of specific mutation configurations and the risk of AP.

### 3.7. CPA1 Mutations in Correlation with a Specific Etiological Factor

An important aspect is to study the effect of the mutation in relation to the clinically defined etiological factor. As mentioned earlier, one of the most frequently diagnosed etiological factors of pancreatitis is alcohol; however, not all alcoholics develop pancreatitis. The question therefore arises as to what factors influence this phenomenon and whether genetic predispositions exist that protect against disorders of pancreatic homeostasis despite the influence of unfavorable factors such as alcohol. This aspect is often discussed in the world literature. Research conducted by Cichoż-Lach H. et al. [32] did not confirm the relationship between mutations in the *CTRC* genes (p.Trp55*, p.Arg254Trp, and c.738_761del) and the risk of alcohol-related chronic pancreatitis in the Polish population. Research led by Ní Chonchubhair H.M. [33] showed a relationship between the occurrence of the *SPINK1* p.Asn34Ser mutation in the Irish population; however, both alcoholic CP and CP with an unspecified etiological factor were analyzed in the study group. Masamune et al. [34] also found a lack of association between the *SPINK1* p.Asn34Ser mutation and alcohol-induced CP while emphasizing the important role of mutations in non-alcoholic CP. Rosendahl, J. et al. [35] released a publication in 2017 presenting the GWAS (genome-wide association study) research on the largest European population to date—in which healthy people who do not abuse alcohol as well as people who abuse alcohol but do not have signs of CP were selected as a control group—and determined the relationship between the *ADH1B* mutation p.His48Arg (c.143A>G) and the risk of alcohol-related CP. The relationship between mutations in the *CTRB1-CTRB2* locus and the risk of CP was also determined and was statistically significant compared to both control groups. Studies conducted by Witt et al. [18] that determined the relationship between mutations in *CPA1* and the risk of CP were carried out in a group of patients with non-alcoholic CP. So far, there are no references in the literature regarding the relationship between *CPA1* mutations and the risk of alcohol-induced CP or AP. In this study, no relationship was observed between alcohol-induced AP and mutations in *CPA1*. This lack of dependence may be due to the absence of a control group consisting of people who abuse alcohol but did not develop AP. Analyzing mutations in this particular group would make it possible to identify markers of susceptibility to the onset of the disease depending on the environmental factor of alcohol consumption. From the perspective of pancreatitis, prevention identifying these mutations could serve as the basis for implementing lifestyle changes in patients.

### 3.8. Genetic Mutations in the Aspect of Family Burden

Observations on the occurrence of diseases in more than one family member are extremely important. Numerous analyses in the literature focus on familial pancreatitis, for which many significant genetic mutations have been successfully described, including those in the *PRSS1* and *SPINK1* genes. In this study, data on family history were collected in 258/301 patients with acute pancreatitis, of which positive family history was recorded in 35 cases. There was no direct relationship between a positive family history and the occurrence of mutations (Fisher’s test). In the case of the *CPA1*(NM_001868.4)c.622G>A (p.Ala208Thr) mutation, statistical significance was not demonstrated; however, the mutation was present only in the group of patients with no positive family history (18/221 vs. 0/35). Presumably, the mutation may be relevant to the development of inflammation in the presence of environmental factors, rather than an inherited predisposition.

Summarizing the mechanisms of induction of the inflammatory process in the pancreas associated with genetic mutations can be based on four key pillars. The first increased conversion of trypsinogen to trypsin (*PRSS1* mutations); the second disorder in the mechanism protected against trypsinogen activation (*SPINK1* and *CTRC* mutations), the third disorder in homeostasis was caused by a change in the pH of pancreatic juice (*CFTR*); the fourth was the occurrence of endoplasmic reticulum stress due to accumulation of misfolded proteins (*CPA1*) [19,36,37]. Ramsey, M.L. et al. used the germline multigene panel including *CASR, CFTR, CPA1, CTRC, PRSS1,* and *SPINK1* genes to analyze the prevalence of pathogenic variants in acute and chronic pancreatitis with respect to ethnicity and family history. There were no cases of *CPA1* pathogenic variants in both the control and AP groups but it should be mentioned that the authors did not analyze the variants with uncertain significance (VUS). According to the clinical database (NCBI), there are only seven variants in *CPA1* classified as pathogenic/likely pathogenic [38]. In this study, several new mutations of the *CPA1* gene were identified, and the relationship between their incidence and the risk of developing acute pancreatitis was determined. The frequencies of mutations in the population of the Świętokrzyskie Province were compared with the European and Chinese populations and mutations characteristic for the studied population were selected. The results obtained indicate a relationship between the occurrence of mutations in the *CPA1* gene and the risk of AP.

### 3.9. Animal Models

An unambiguous assessment of the effect of mutations on the occurrence of the disease is possible through studies using an animal model. Mutations causing a change in the amino acid sequence can lead to the accumulation of misfolded synthesized carboxypeptidase can activate an inflammatory response in the pancreas. In 2019, Hegyi E. and Sahin-Toth M. [28] studied in vivo the effect of the *CPA1* p.Asn256Lys mutation on the risk of CP in mice. These were the first studies in a mouse model to determine the effect of the misfolded carboxypeptidase protein on the risk of CP and confirm the possibility of inflammation via ERS. In the studies published by Hegyi E. and Sahin-Toth M, it was clearly shown that mice carrying the *CPA1* p.Asn256Lys mutation developed chronic pancreatitis and the level of tissue damage increased over time; therefore, after three months, the pancreatic tissue was already damaged 15%, after 6 months, the damage covered 20% of the tissues, while after a year, about 30% of the pancreatic tissue was damaged, which indicates a high potential of the *CPA1* gene mutation as a factor increasing the risk of chronic pancreatitis [28]. The mouse model clearly indicates a significant role of the mutation in the process of inflammation, and the studies conducted in this work confirm the relationship between the occurrence of mutations in the *CPA1* gene and the risk of developing an acute form of the disease. The most important scientific achievement of this research is the delivery of new data on genetic variability in the group of AP patients as well as the determination of potential genetic factors predisposing to the development of inflammation and complications after AP.

### 3.10. Study’s Limitations

This study was conducted using the Sanger sequencing of selected regions of the *CPA1* gene. For potential genetic marker identification and a more comprehensive view of the mutation landscape, the Next-Generation Sequencing (NGS) platform could be more useful. It offers high throughput and enables the study of a larger number of individuals, facilitating the identification of both rare and common variants. In addition, this study was carried out on a population with a high incidence of AP [1], however, we intend to increase the number and diversity of the study group.

## 4. Materials and Methods

This study included 320 patients with a history of pancreatitis and 184 healthy volunteers (control group). Peripheral blood was collected from both groups in EDTA tubes and deposited in the Department of Interventional Medicine with the Laboratory of Medical Genetics, Collegium Medicum of the Jan Kochanowski University. Clinical data of patients with pancreatitis included etiology of inflammation and family history. The material for this study was collected from participants between 2013 and 2020. The characteristics of the study group are presented in Table 1. The etiology of acute pancreatitis was divided into 6 categories: alcoholic, biliary, idiopathic, drug-induced, hypertriglyceridemia, and acute pancreatitis in the postpartum period. Data collected in the family history concerned the incidence of pancreatic diseases and dysfunction in general (diabetes, pancreatitis, pancreatic cancer, etc.).

Before isolation, the blood samples were de-frosted and gently mixed to homogenize the input material. Genomic DNA was extracted from a volume of approx. 200 using a kit for DNA isolation from peripheral blood Genomic Micro AX Blood Gravity (A&A Biotechnology, Gdańsk, Poland) according to the manufacturer’s protocol. Absorbance measurements at 260/280 were used to check the purity and quantify the concertation [ng/µL] of the isolates using DeNovix Ds-11 (DeNovix Inc., Wilmington, NC, USA). The minimum DNA concentration required for the analysis was 10 ng/µL. The material was stored at 4 °C.

To amplify selected Carboxypeptidase A1 gene (*CPA1*) fragments, PCR was performed using the commercially available DreamTaq Green PCR Master Mix (2X) (Thermo Scientific™, Waltham, MA, USA), according to the manufacturer’s protocol, with 10 pmol forward- and reverse-primers designed by Witt et al. [18] and 2 μL DNA filled with MiliQ water for a total volume of 25 μL of solution. DNA amplification was performed using Master Cycler X50 (Eppendorf SE, Hamburg, Germany) according to the following cycling conditions: denaturation at 95 °C for 3 min, followed by 35 cycles of denaturation at 95 °C for 20 s, annealing at 62 °C for 40 s, and extension at 72 °C for 2 min, followed by a final extension at 72 °C for 6 min. After electrophoresis on a 2% agarose gel with the addition of the Syngen Green DNA Gel Stain dye (Syngen Biotech Sp. z o.o., Wrocław, Poland), the PCR products were visualized and stored at 4 °C until sequencing but for no longer than 48 h. Sequencing of the amplified DNA fragments was performed by Macrogen (Amsterdam, The Netherlands). Samples were transported at 4–8 °C and then pre-purified and sequenced. The quality criteria of the chromatograms were in accordance with ACGS (Association for Clinical Genetic Science) guidelines [39]. FinchTV version 1.4.0 for Windows (Geospiza) software and The Quality Check App (ThermoFisher, Waltham, MA, USA, available on https://apps.thermofisher.com/apps/spa/#/apps accessed on 11 October 2023) were used for the analysis. The quality of the chromatograms was also assessed based on the reading intensity for the entire length of the sequence. It was assumed that the noise value for the entire sequence may not exceed 5% of the intensity of the main readings. QV (quality values) were determined with the assumption that for a single reading, the values must be in the range of QV = 20–50, and for mixed readings (as in the case of heterozygotes), the range QV = 10–50 applies. Next, the CRL value was analyzed. The length of continuous reading for the conducted analytes varied depending on the analysis, and for the fragment covering the sequences of exons 5–6, a CRL greater than 500 was assumed, while for the analysis of the fragment covering the sequences of exons 7–9, a CRL greater than 960 was assumed.

The sequences were compared with the reference sequence for the carboxypeptidase A1 gene available in the NCBI database, International Sequence Number (OMIM: 114850; NM_001868.4). Sequence comparison to reference sequences was performed using the Basic Local Alignment Search Tool software (https://blast.ncbi.nlm.nih.gov/Blast.cgi, BLAST, NCBI, USA accessed on 15 November 2023). Homo- and heterozygous changes were determined according to their location in the reference sequence, with identified loci marked using the notation “g” followed by the position of the nucleotide. Nucleotide substitutions are denoted according to the generally prevailing nomenclature using the sign “>”, insertions are “ins”, and deletions are “del”. For changes localized in exons, the influence of mutations on the resulting protein was additionally determined. All mutations were checked against the international database ClinVar (NCBI) and Varsome Clinical for reference numbers (RefSNP, rs).

Statistical analysis: Qualitative data distributions were delineated by frequency and percentages. Frequency comparisons were conducted using the chi-square test or Fisher’s exact test. The normality of the distribution of quantitative features was assessed with the Shapiro–Wilk test. As the assumption of normality was breached, the distributions of quantitative features were compared using the Mann–Whitney U test. All statistical tests performed were two-sided. The criterion of statistical significance was *p* < 0.05. The R program, R Core Team version 3.6.2 (2019) was used in the calculations. R: A language and environment for statistical computing. R Foundation for Statistical Computing, Vienna, Austria. URL https://www.R-project.org/.

## 5. Conclusions

In this work, we highlighted the potential role of *CPA1* variants in acute pancreatitis risk. The *CPA1* gene is characterized by high sequence variability and regions in which mutations increase the risk of AP. So far, several genes with potential application in the diagnosis of pancreatitis have been described, including *PRSS1*, *CFTR*, *CTRC*, and *SPINK1*. Despite these findings, there is no genetic panel available that would enable genetic screening tailored to specific populations. Creating a screening panel will only be possible by using new analytical techniques—mainly whole genome sequencing (WGS)—analysis of the co-occurrence of environmental factors, assessment of the impact of epigenetic mechanisms, and gene regulation. It is necessary to conduct research on a larger population, which would also enable genome-wide association studies (GWAS). This research was carried out on a study group from a region with a high incidence of AP, therefore, it is important to extend the analysis to more diverse populations [1]. It should be emphasized that this research was carried out due to the increasing number of people suffering from AP, especially those of idiopathic etiology.

## Figures and Tables

**Table 1:** Characterization of study groups.

	Acute Pancreatitis (n = 301)	Controls (n = 184)
Male (%)	174 (57.8%)	75 (40.8%)
Female (%)	127 (42.2%)	109 (59.2%)
Mean Age	54.5	51.2
Etiology (male/female)		
biliary	133 (45/88)	N/A
alcoholic	100(90/10)	
idiopathic	62 (36/26)	
postpartum period	2 (0/2)	
hypertriglyceridemia	2 (2/0)	
drug-induced	2 (1/1)	
Familial history		nd
Pancreatic diseases in family	35	
No cases	221	
nd	45	

N/A—not applicable, nd—no data.

**Table 2:** CPA1(NM_001868.4) variants detected in AP and controls with corresponding effect (Fisher’s exact test, two-tailed). OR—odds ratio, RR—relative risk.

CDS	Aa Change		Controls (n = 184)	AP (n = 301)	*p*	OR [95% CI]	RR [95% CI]
c. 625A>T	p. Ile209Phe	rs782417364	25 (13.6%)	16 (5.3%)	*	0.36 [0.19–0.69]	0.39 [0.22–0.72]
c.987 + 57G>A	-	-	10 (5.4%)	39 (13.0%)	**	2.59 [1.26–5.32]	2.38 [1.22–4.66]
c.1072 + 84del	-	-	0 (0.0%)	7 (2.3%)	*	N/A	N/A
c.1072 + 94del	-	-	9 (4.9%)	4 (1.3%)	*	0.26 [0.08–0.87]	0.27 [0.08–0.09]
region c.484 − 33; c.585 + 48	-	-	3 (1.6%)	22 (7.3%)	**	4.75 [1.4–16.13]	4.48 [1.36–14.77]

RefSeq: *CPA1*(NM_001868.4); * *p* = 0.05–0.011; ** *p* < 0.01; N/A—not applicable.

**Table 3:** Variants identified in *CPA1*(NM_001868.4) with occurrence in both groups (Fisher’s exact test, two-tailed).

CDS	Aa Change		Controls (n = 184)	AP (n = 301)	*p*
c.484 − 34del	-	-	3 (1.6%)	0 (0.0%)	0.0541
c.622G>A	p.Ala208Thr	rs34474469	21 (11.4%)	20 (6.6%)	0.067
c.925T>C	p.Ser309Pro	-	3 (1.6%)	0 (0.0%)	0.0541
c.938T>G	p.Met313Arg	-	3 (1.6%)	0 (0.0%)	0.0541
c.987 + 98A>T,G,C	-	-	8 (4.3%)	4 (1.3%)	0.0657
c.1072 + 76del	-	-	2 (1.1%)	12 (4.0%)	0.0642
c.1072 + 99del	-	-	4 (2.2%)	1 (0.3%)	0.0711

RefSeq: *CPA1*(NM_001868.4).

## Data Availability

The original contributions presented in this study are included in the article/Supplementary Materials, further inquiries can be directed to the corresponding author.

## Supplementary Materials

The following supporting information can be downloaded at: https://www.mdpi.com/article/10.3390/ijms252011301/s1.
