# Supplementary material for: Genetic Variability in the CPA1 Gene and Its Impact on Acute Pancreatitis Risk: New Insights from a Large-Scale Study
Source: Int J Mol Sci. 2024 Oct 21;25(20):11301. doi: 10.3390/ijms252011301 (PMC11508624; doi:10.3390/ijms252011301)
Supplement: Supplementary file 1 [file ijms-25-11301-s001.zip › ijms-3265688-supplementary.pdf]

Table S1. Sumarrized List of mutations localized in the coding regions of CPA1(NM_001868.4) gene.

CDS change	Protein change	
c.494G>C	p.Gly165Ala	rs200317425
c.465G>C	p.Gly165=	-
c.497G>A	p.Gly166Asp	rs144546424
c.499A>G	p.Ser167Gly	-
c.501T>C	p.Ser 167=	-
c.511G>A	p.Ala171Thr	rs1554411487
c.515T>A	p.Ile172Asn	-
c. 516C>A	p.Ile172=	-
c. 517T>A	p.Trp 173Arg	-
c.524 A>T	p.Asp175Val	-
c.530G>T	p.Gly177Val	-
c.531C>G	p.Gly177=	rs1051725707
c.532A>G	p.Ile178Val	-
c.548G>C	p.Trp183Ser	-
c.559G>C	p.Ala187Pro	rs1554411500
c.574 T>A	p.Phe192Ile	-
c. 577A>C	p. Ala193=	-
c.585G>A	p.Lys195=	-
c.588C>A	p.196 Ile=	-
c.594 A>G	p.198Gln=	-
c.594 A>T	p.Gln198His	-
c.595 G>T	p.Asp199Tyr	
c.598T>A	p.Tyr200Asn	rs1562967649
c.599_600delACinsG	p.(Tyr200TrpfsTer91)	rs1584851947
c.600 C>G	p.Tyr200Ter	rs12706927
c.601 G>A	p.Gly201Arg	rs782234205
c.604 C>A	p.Gly201Lys	-
c.609 T>G	p.Asp203Glu	-
c.622G>A	p.Ala208Thr	rs34474469
c.625A>T	p. Ile209Phe	
c.625A>C,	p.Ile209Leu	
c.631G>A,	p.Asp211Asn	-
c.634 A>T	p.Thr212Ser	-
c. 640G>A,	p.Asp214Asn	-
c. 647T>C,	p.Phe216Ser	-
c.650T>A,	p.Leu217Gln	-
c.651G>C,	p.Leu217=	-
c.658G>A,	p.Val220Ile	rs1554411595
c.665A>G,	p.Asn222Ser	-
c.669 T>G,	p.Pro223=	-
c.670G>A,	p.Asp224Asn	-
c.674G>A	p.Gly225Asp	-
c.695C>G,	p.Thr232Arg	rs114488732
c.791C>G	p.Ser264Cys	-
c.792C>G	p.Ser264=	rs782313134
c.798C>G	p.Ala266=	-
c.800G>C	p.Ser267Thr	-

c.801C>A	p.Ser267Arg	-
c.803G>A	p.Ser268Lys	-
c.811T>G	p.Cys271Gly	-
c.813C>G	p.Cys271Trp	-
c.816G>T	p.Ser 272=	rs34843162
c.822T>A	p.Thr274=	-
c.837T>C	p.Phe279=	-
c.847G>A	p.Glu283Lys	rs782561684
c.859A>G	p.Lys287Glu	rs550709882
c.871G>A	Asp291Asn	-
c.878T>G	p.Val293Gly	-
c.885C>A	p.Asp295Glu	-
c.886C>T	p.His296Tyr	
c.886C>G	p.His296Val	
c.891 G>A	p.Gly297=	-
c.895A>G	p.Ile299Val	rs533352347
c.898A>G	p.Lys300Gly	-
c.899A>G	p.Lys300Arg	-
c. 902C>G	p.Ala301Gly	-
c.913 A>T	p.Ile305Phe	-
c.914 T>G	p.Ile305Ser	-
c.919A>G	p.Ser307Gly	-
c.922 T>A	p.Tyr308Asn	-
c.925T>C	p.Phe309Leu	-
c.930G>C	p.Gln310His	-
c.932T>C	p.Leu311Pro	-
c.938T>g,	p.Met313Arg	-
c.938T>A	p.Met313Lys	-
c.982G>A	p.Glu328Lys	rs1554411843
c.984 G>A	p.Glu328=	-

CDS – coding DNA sequence.

Table S2. Genotype frequencies of CPA1(NM_001868.4) *c.622G>A* in respect of familial history, Fischer Exact test, two tailed.

		**No cases in the family n=221**	**Positive familial history n=35**	***p***
c.622G>A [p.Ala208Thr]	G/G	203	35	
	G/A	17	0	G/G vs. G/A+A/A *p*>0,05
	A/A	1	0	G/G + G/A vs. A/A *p*>0,05
	G	423	70	*p*>0,05
	A	19	0	
